# In Vitro Inhibition of Human UDP-Glucuronosyl-Transferase (UGT) Isoforms by Astaxanthin, β-Cryptoxanthin, Canthaxanthin, Lutein, and Zeaxanthin: Prediction of in Vivo Dietary Supplement-Drug Interactions

**DOI:** 10.3390/molecules21081052

**Published:** 2016-08-12

**Authors:** Yu Fen Zheng, Jee Sun Min, Doyun Kim, Jung Bae Park, Sung-Wook Choi, Eun Seong Lee, Kun Na, Soo Kyung Bae

**Affiliations:** 1 College of Pharmacy, Integrated Research Institute of Pharmaceutical Sciences, The Catholic University of Korea, Bucheon 14662, Korea; cathy8521@hotmail.com (Y.F.Z.); sunny08@catholic.ac.kr (J.S.M.); doyun325@catholic.ac.kr (D.K.); sacramentou@naver.com (J.B.P.); 2 Department of Biotechnology, The Catholic University of Korea, Bucheon 14662, Korea; choisw@catholic.ac.kr (S.-W.C.); eslee@catholic.ac.kr (E.S.L.); kna6997@catholic.ac.kr (K.N.)

**Keywords:** xanthophylls, β-cryptoxanthin, in vitro UGTs inhibition, in vitro-in vivo extrapolation

## Abstract

Despite the widespread use of the five major xanthophylls astaxanthin, β-cryptoxanthin, canthaxanthin, lutein, and zeaxanthin as dietary supplements, there have been no studies regarding their inhibitory effects on hepatic UDP-glucuronosyltransferases (UGTs). Here, we evaluated the inhibitory potential of these xanthophylls on the seven major human hepatic UGTs (UGT1A1, UGT1A3, UGT1A4, UGT1A6, UGT1A9, UGT2B7 and UGT2B15) in vitro by LC-MS/MS using specific marker reactions in human liver microsomes (except UGT2B15) or recombinant supersomes (UGT2B15). We also predicted potential dietary supplement-drug interactions for β-cryptoxanthin via UGT1A1 inhibition. We demonstrated that astaxanthin and zeaxanthin showed no apparent inhibition, while the remaining xanthophylls showed only weak inhibitory effects on the seven UGTs. β-Cryptoxanthin mildly inhibited UGT1A1, UGT1A3, and UGT1A4, with IC_50_ values of 18.8 ± 2.07, 28.3 ± 4.40 and 34.9 ± 5.98 μM, respectively. Canthaxanthin weakly inhibited UGT1A1 and UGT1A3, with IC_50_ values of 38.5 ± 4.65 and 41.2 ± 3.14 μM, respectively; and lutein inhibited UGT1A1 and UGT1A4, with IC_50_ values of 45.5 ± 4.01 and 28.7 ± 3.79 μM, respectively. Among the tested xanthophyll-UGT pairs, β-cryptoxanthin showed the strongest competitive inhibition of UGT1A1 (K_i_, 12.2 ± 0.985 μM). In addition, we predicted the risk of UGT1A1 inhibition in vivo using the reported maximum plasma concentration after oral administration of β-cryptoxanthin in humans. Our data suggests that these xanthophylls are unlikely to cause dietary supplement-drug interactions mediated by inhibition of the hepatic UGTs. These findings provide useful information for the safe clinical use of the tested xanthophylls.

## 1. Introduction

Xanthophylls have been studied for their beneficial effects, including their powerful antioxidant activities and association with lower incidence of chronic diseases [1,2,3,4]. Many xanthophylls, especially astaxanthin (AS), β-cryptoxanthin (βC), canthaxanthin (CA), lutein (LU) and zeaxanthin (ZE), are widely used in clinical practice. AS is found mainly in seafood, such as crustaceans, salmonids, and farmed fish feed; and owing to its strong antioxidant activity, it has been used to protect against cardiovascular problems, diabetes, chronic inflammatory diseases, various cancers, and some immunological diseases [5,6,7]. βC is mainly found in fruits and vegetables, such as tangerines, green grapes, coriander, parsley, basil, red peppers, and pumpkin, and it has been shown to have antioxidant activity [8,9]. In addition, intake of CA has been associated with a reduced risk of cancer [10,11]. LU and ZE are the two major xanthophylls found in the human macula and retina [12,13]. In the human diet, the highest concentrations of LU are found in dark green leafy vegetables, corn, and egg yolks, while ZE is found in corn, orange pepper, oranges, and tangerines [14]. Intake of LU and ZE was associated with decreased age-related macular degeneration risk and less visual impairment [12,13,15].

Owing to the beneficial health effects of xanthophylls, their use as dietary supplements has been rapidly growing [2]. The increasing use of dietary/herbal supplements presents a special challenge when managing patients′ health due to the risk for potential dietary/herbal supplement-drug interactions [16,17]. Recent surveys revealed that 25% of dietary/herbal supplement users take one or more prescription drugs [18,19]. Many dietary/herbal supplement-drug interactions involve the inhibition or induction of drug-metabolizing enzymes, resulting in altered systemic exposure and subsequent adverse drug reactions or loss of efficacy [16,17,20]. Among the drug-metabolizing enzymes, cytochrome P450 (CYPs) and uridine 5′-diphosphate (UDP)-glucuronosyltransferases (UGTs) are responsible for the metabolic clearance of more than 90% of drugs [21].

Despite the widespread use of xanthophylls, there are few reports of interactions between drug-metabolizing enzymes and AS, βC, CA, LU and ZE. Gradelet et al. [22] reported that AS and CA were substantial inducers of the phase I enzymes CYP1A1 and CYP1A2 and the phase II enzyme 4NP-UGT in rats. In contrast, LU had no effect on phase I or II xenobiotic-metabolizing enzymes activities in rats [22]. In human hepatocytes, AS induced the activity of CYP2B6 and CYP3A4 [23]. In our previous in vitro study, we confirmed that these five xanthophylls had insignificant effects on nine cytochrome P450s (CYP1A2, CYP2A6, CYP2B6, CYP2C8, CYP2C9, CYP2C19, CYP2D6, CYP2E1 and CYP3A4/5) in human liver microsomes [24]. However, the inhibitory effects of these five xanthophylls (AS, βC, CA, LU and ZE) on UGT activities have not been reported.

UGTs metabolize many endogenous substances (e.g., bilirubin, steroid hormones, thyroid hormones, bile acids and fat-soluble vitamins) as well as numerous xenobiotics (e.g., drugs, chemical carcinogens and dietary substances) [25,26,27]. Thus, inhibition of UGT-mediated reactions could not only induce metabolic disorders but also result in serious drug-drug interactions. Recently, 22 human UGTs were identified based on sequence homology, which are generally categorized into three major subfamilies (UGT1A, 2A and 2B) [27]. The liver is the major site of UGTs, and UGT1A1, 1A3, 1A4, 1A6, 1A9, 2B7 and 2B15 are considered to be the most important human liver drug-metabolizing isoforms [28,29].

The aim of this study was to investigate the inhibitory potential of five commonly used xanthophylls (AS, βC, CA, LU and ZE) on the seven major human hepatic UGT isozymes (UGT1A1, UGT1A3, UGT1A4, UGT1A6, UGT1A9, UGT2B7 and UGT2B15) in vitro and to quantitatively predict their potential for dietary supplement-drug interactions in vivo.

## 2. Results

### 2.1. Inhibitory Effects of Five Xanthophylls on the Major UGT Isoforms in Human Liver Microsomes

**Figure 1:**
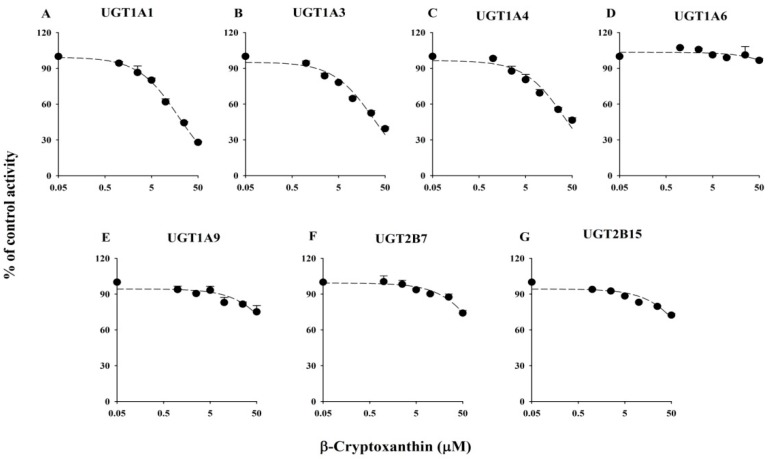
IC_50_ curves of β-cryptoxanthin for human UGTs activities including UGT1A1 for β-estradiol-3-glucuronidation (**A**); UGT1A3 for chenodeoxycholic acid 24-acyl-β-d-glucuronidation (**B**); UGT1A4 for trifluoperazine-*N*-glucuronidation (**C**); UGT1A6 for serotonin-*O*-glucuronidation (**D**); UGT1A9 for propofol-*O*-glucuronidation (**E**); and UGT2B7 for zidovudine-5′-glucuronidation (**F**) in human liver microsomes, and UGT2B15 for 4-methylumbelliferyl glucuronidation (**G**) in recombinant human UGT2B15 supersomes. Data are the mean ± standard deviation of triplicate determinations. The dashed lines represent the best fit to the data using non-linear regression.

The inhibitory effects of AS, βC, CA, LU, and ZE on the activity of six UGTs are illustrated in Figure 1 (βC) and Supplementary Figures S1–S4 (AS, CA, LU and ZE, respectively). The IC_50_ values of the positive controls and five xanthophylls are shown in Table 1. The IC_50_ values of the known potent inhibitors used in the inhibition studies were similar to previously reported values. The five tested xanthophylls showed only weak inhibitory effects on the six UGTs. βC mildly inhibited UGT1A1, UGT1A3, and UGT1A4, with IC_50_ values of 18.8 ± 2.07, 28.3 ± 4.40, and 34.9 ± 5.98 μM, respectively, and showed no apparent inhibition of UGT1A6, UGT1A9, and UGT2B7 (Table 1). CA weakly inhibited UGT1A1 and UGT1A3, with IC_50_ values of 38.5 ± 4.65 and 41.2 ± 3.14 μM, respectively, and LU weakly inhibited UGT1A1 and UGT1A4, with IC_50_ values of 45.5 ± 4.01 and 28.7 ± 3.79 μM, respectively. AS and ZE had no apparent inhibitory effects on any of the six tested liver UGTs; the residual enzyme activities at the highest tested concentration (50 μM) were greater than 80%, except for UGT1A1 (61.2%) and UGT1A3 (64.2%) in the presence of ZE (Table 1). Among the five tested xanthophylls, βC showed the lowest IC_50_ value (18.8 ± 2.07 μM) for UGT1A1.

### 2.2. Inhibitory Effects of Five Xanthophylls of UGT2B15 Activity

The inhibitory effects of AS, βC, CA, LU, and ZE on UGT2B15 activity in UGT2B15 supersomes are shown in Figure 1 and Supplementary Figures S1–S4, respectively. The IC_50_ values of the positive control and five xanthophylls are listed in Table 1. No selective inhibitor probes for UGT2B15 have been identified; thus, we used amitriptyine as a positive control, although it is not specific for the UGT2B15 [30], to evaluate the suitability of these experiments. None of the five tested xanthophylls inhibited UGT2B15 activity, as the residual activities at 50 μM were all greater than 70%.

### 2.3. K_i_ Determination of βC on the UGT1A1 Activity

**Figure 2:**
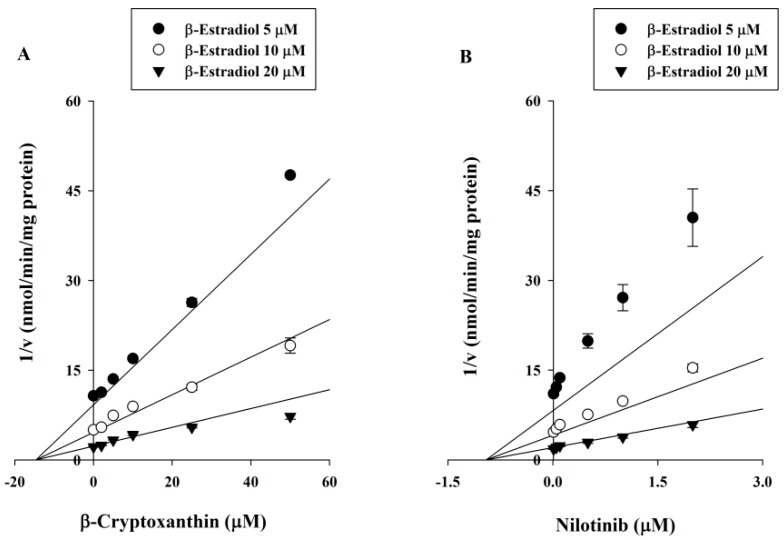
Dixon plots of the inhibitory effects of β-cryptoxanthin (**A**) and nilotinib (**B**) against UGT1A1-catalyzed β-estradiol-3- glucuronide in human liver microsomes. The concentrations of β-estradiol were determined 5 (●), 10 (○), and 20 (▲) μM, respectively. The v represents formation rate of β-estradiol-3-glucuronidation (nmol/min/mg protein). Data are the mean ± standard deviation of triplicate experiments. The solid lines of β-cryptoxanthin and nilotinib fit well with a competitive mode of inhibition.

To further characterize the inhibition of UGT1A1 by βC, enzyme kinetic experiments were performed in the presence of various concentrations of βC and β-estradiol. Samples containing the known potent UGT1A1 inhibitor nilotinib were included in the analysis. Representative Dixon plots showing UGT1A1 inhibition by βC and nilotinib in human liver microsomes are shown in Figure 2. Based on the nonlinear regression analysis, βC showed competitive inhibition against UGT1A1-catalyzed β-estradiol-3-glucuronidation, with a K_i_ of 12.2 ± 0.985 μM. Nilotinib showed stronger competitive inhibition of UGT1A1, with a K_i_ of 0.811 ± 0.126 μM.

## 3. Discussion

In this study, the inhibitory effects of five xanthophylls (AS, βC, CA, LU, and ZE) on the seven major human hepatic UGT isozymes (UGT1A1, UGT1A3, UGT1A4, UGT1A6, UGT1A9, UGT2B7, and UGT2B15) were evaluated in vitro using human liver microsomes (all except UGT2B15) or recombinant supersomes (UGT2B15). The difference between human recombinant supersomes and human liver microsomes is that the former contain a single UGT enzyme, whereas the latter contain all hepatic UGT isoforms. Therefore, human liver microsomes are closer to the in vivo environment due to the presence of all other UGT enzymes. UGTs may form heterodimers, as was reported for some isoforms, which may affect enzyme activity [31]. Because our goal was to screen for interactions that may have clinical significance, the use of human liver microsomes was more appropriate. This was possible because selective substrates are available for the six of the UGT enzymes in human liver microsomes [32,33]. However, because authentic *S*-oxazepam, a typical substrate of UGT2B15, and the corresponding glucuronide are not available, we used recombinant UGT2B15 supersomes and the nonspecific substrate 4-methylumbelliferone to evaluate UGT2B15 activity, as previously described [34].

The current findings demonstrated that AS, βC, CA, LU, and ZE had only weak inhibitory effects on the seven UGTs examined. AS and ZE did not inhibit any of the seven UGTs. βC mildly inhibited UGT1A1, UGT1A3, and UGT1A4; CA weakly inhibited UGT1A1 and UGT1A3; and LU weakly inhibited UGT1A1 and UGT1A4 (Table 1). Inhibition of UGT1A1, UGT1A3, and/or UGT1A4 by these five xanthophylls was not unexpected based on an amino acid sequence analysis [27]. UGT1A3 and UGT1A4 share 93% amino acid sequence identity, and they share 71% homology with UGT1A1. The high sequence similarity between UGT1A1, UGT1A3, and UGT1A4 may indicate similar three-dimensional structures and similar binding to these five structurally related xanthophylls. Among the tested xanthophylls, βC most strongly inhibited UGT1A1 (IC_50_ = 18.8 ± 2.07 μM and K_i_ = 12.2 ± 0.985 μM) in a competitive manner, although it is a much less potent inhibitor than nilotinib (K_i_ = 0.811 ± 0.126 μM). UGT1A1 is responsible for the metabolism of several endogenous and exogenous substrates, including 15% of the top 200 drugs used in the United States in 2002 [35] that have glucuronidation as a clearance mechanism. UGT1A1 inhibition is particularly important for any drug with a narrow therapeutic index, such as etoposide or irinotecan [36,37].

As far as we know, for reversible inhibitors, the magnitude of the drug-drug interaction is affected by both the in vitro inhibition constant (K_i_) and the in vivo inhibitor concentration. For the inhibition of UGT1A1 by βC, the magnitude of the in vivo drug-drug interaction is given by the ratio of the area under the plasma drug concentration-time curve in the presence and absence of the inhibitor (AUC_i_/AUC), as previously reported:

AUC_i_/AUC = 1 + [I]_in vivo_/K_i_ (1)

where [I]_in vivo_ is the in vivo concentration of βC. In this study, the maximum plasma concentration (C_max_) of βC was used as the [I]_in vivo_ value, and the fraction metabolized by the UGT1A1 of the co-administrated drug was supposed to be 1. A pharmacokinetic study found that, for a single-dose carotenoid supplement containing 1.3 mg of esterified βC in healthy subjects, the mean C_max_ was approximately 0.15 μM [8]. Using this C_max_ value, the AUC_i_/AUC ratio was calculated as 1.012 for UGT1A1, indicating that the AUC of the co-administered drug may increase 1.2% when βC is co-administered with clinical drugs that undergo 100% UGT1A1-mediated elimination. Thus, our data strongly suggest that βC is unlikely to cause a clinically significant metabolic drug-drug interaction via inhibition of any of the seven major human hepatic UGTs involved in drug metabolism.

This study has however some limitations. First, the final concentration of organic solvent in the human liver microsome incubation mixture was 2.5% because the five xanthophylls had very low solubilities. It has been reported that higher organic solvent concentrations are associated with stronger inhibition of UGT activity in human liver microsomes [34], thus, the organic solvent concentrations must be kept low (<1%) to avoid any effects on microsomal activity. Second, the in vitro-in vitro extrapolation of human clearance is improved by addition of bovine serum albumin (BSA) to an incubation mixture [38,39]. BSA sequesters inhibitory long-chain fatty acids, thereby increasing the unbound intrinsic clearance for UGT1A9 and 2B7 [38,39]. Thus, the effects of BSA addition should be evaluated for future studies. Finally, another potential problem is that our calculation is based only on the inhibition of hepatic UGT1A1 by βC, and UGT1A1 is also expressed in the gastrointestinal tract. Thus, these UGTs may be inhibited in the small intestine, and the concentrations of inhibitors in the intestine may differ from those used to predict the AUC ratio. Thus, we should be cautious when extrapolating the in vitro data to in vivo drug interactions.

To the best of our knowledge, there have been no previous reports examining the inhibitory potential of dietary supplement-drug interactions for the five main xanthophylls and major hepatic UGT isozymes. Based on the in vitro-in vivo extrapolation, the data presented here strongly suggest that these five xanthophylls are unlikely to cause clinically significant metabolic dietary supplement-drug interactions through inhibition of the major drug-metabolizing hepatic UGT isoforms. These findings improve our understanding of xanthophyll-drug interactions and predict safe use for these five xanthophylls in clinical practice.

## 4. Experimental Section

### 4.1. Materials and Reagents

Pooled human liver microsomes from equal gender mix from 150 donors and a recombinant human UGT2B15 supersome were purchased from Corning Life Sciences (Woburn, MA, USA). The human liver microsomes studies were performed in accordance with the Declaration of Helsinki and designated exempt from review by the Catholic University of Korea Institutional Review Board. Astaxanthin (AS), β-cryptoxanthin (βC), canthaxanthin (CA), lutein (LU), zeaxanthin (ZE), alamethicin, amitriptyline, β-estradiol, β-estradiol-3-glucuronide, chenodeoxycholic acid, diclofenac, 4-methylumbelliferone, 4-methylumbelliferyl glucuronide, trifluoperazine dihydrochloride, serotonin hydrochloride, zidovudine, hecogenin, niflumic acid, chlorpropamide, theophylline, MgCl_2_, alamethicin, and uridine 5′-diphosphoglucuronic acid trisodium salt (UDPGA) were purchased from Sigma‒Aldrich Corporation (St. Louis, MO, USA). Efavirenz, nilotinib, propofol, propofol-*O*-glucuronide, serotonin-*O*-glucuronide, trifluoperazine-*N*-glucuronide, zidovudine-5′-glucuronide, and chenodeoxycholic acid 24-acyl-β-d-glucuronide were purchased from Toronto Research Chemicals (North York, ON, Canada). Deoxyschizandrin (purity: 97.7%) was kindly donated by Dr. Young-Won Chin (Dongguk University, Ilsan, Korea). All solvents were of high-performance liquid chromatography (HPLC) grade and were obtained from Burdick & Jackson Company (Morristown, NJ, USA); other chemicals were of the highest quality available.

### 4.2. Inhibitory Effects of Five Xanthophylls on the Major UGT Isoforms in Human Liver Microsomes

The inhibitory effects of AS, βC, CA, LU and ZE on UGT1A1, UGT1A3, UGT1A4, UGT1A6, UGT1A9 and UGT2B7 were evaluated using pooled human liver microsomes, as previously described [32,40]. The formation activities of β-estradiol-3-glucuronide, chenodeoxycholic acid 24-acyl-β-d-glucuronide, trifluoperazine-*N*-glucuronide, imipramine*-N*-β-d-glucuronide, serotonin-*O*-glucuronide, propofol-*O*-glucuronide, and zidovudine-5′-glucuronide were determined to probe the activities of UGT1A1, UGT1A3, UGT1A4, UGT1A6, UGT1A9 and UGT2B7, respectively, in human liver microsomes. The selected concentrations of UGT probe substrates were close to the reported K_m_ values [28,30,41,42,43], thus: 10 μM β-estradiol (UGT1A1), 15 μM chenodeoxycholic acid (UGT1A3), 4 μM trifluoperazine (UGT1A4), 4000 μM serotonin (UGT1A6), 100 μM propofol (UGT1A9), and 100 μM zidovudine (UGT2B7).

In brief, the 90-µL incubation mixture, including pooled human liver microsomes (final concentration 0.25 mg/mL), 100 mM Tris buffer, 25 µg/mL alamethicin, 5 mM MgCl_2_, the UGT-selective substrates, and five xanthophylls (0–50 μM), were pre-incubated for 30 min on ice to allow formation of alamethicin pores. Reac2345tions were initiated by the addition of 10 μL of UDPGA (5 mM) to a final reaction volume of 0.1 mL and incubated at 37 °C for 60 min (except 30 min for UGT1A9) in a shaking water bath. After incubation, each reaction was stopped by adding 50 μL of ice-cold acetonitrile containing chlorpropamide (300 ng/mL) or theophylline (30 μg/mL or 300 μg/mL) as an internal standard. The mixtures were then centrifuged (13,000 *g* for 15 min at 4 °C) and 5-µL aliquots of the supernatants were injected into an LC-MS/MS system. Known potent inhibitors nilotinib (0–10 μM), deoxyschizandrin (0–100 μM), hecogenin (0–50 μM), diclofenac (0–500 μM), niflumic acid (0–5 μM) and efavirenz (0–200 μM) were included as positive controls for UGT1A1 [41], UGT1A3 [44], UGT1A4 [29], UGT1A6 [34], UGT1A9 [45] and UGT2B7 [42], to evaluate the suitability of these experiments and to compare the 50% inhibitory concentrations (IC_50_ values). All incubations were performed in triplicate, and mean values were used for analysis. 

All substrate and inhibitors were dissolved in methanol (except hecogenin and five xanthophylls which were dissolved in dimethylsulfoxide due to their low solubility) and serially diluted to the required concentrations. The final concentration of organic solvent in each incubation mixture including the control was 2.5% (0.5% of methanol and 2.0% of dimethylsulfoxide) (*v/v*).

### 4.3. Inhibitory Effects of Five Xanthophylls of UGT2B15 Activity

Although *S*-oxazepam is a typical substrate of UGT2B15 of human liver microsomes, authentic *S*-oxazepam and the corresponding glucuronide are not commercially unavailable [46]. Thus, the inhibition of AS, βC, CA, LU, and ZE on the UGT2B15 activity was assessed using 4-methylumbelliferone as the nonspecific probe substrate for all UGTs except UGT1A4 [34] in recombinant human UGT2B15 supersomes.

The inhibitory effects of AS, βC, CA, LU, and ZE on UGT2B15 were assessed in recombinant human UGT2B15 supersomes, as previously described [17,32]. Briefly, 90 µL incubation mixtures, including recombinant human UGT2B15 supersomes (final concentration 0.75 mg/mL), 100 mM Tris buffer, 5 mM MgCl_2_, 4-methylumbelliferone (250 μM), and five xanthophylls (0‒50 μM) were pre-incubated for 5 min at 37 °C. The reaction was initiated by addition of 10 µL 5 mM UDPGA (to 100 µL total volume) followed by incubation for 120 min at 37 °C in a shaking water bath. Reactions were stopped by addition of 50 µL of ice-cold acetonitrile containing chlorpropamide (300 ng/mL) as an internal standard, and they were chilled and centrifuged (13,000 *g*, 15 min, 4 °C). An aliquot of 5 μL of supernatant was injected into an LC-MS/MS system. Additionally, identical parallel incubation samples containing a known UGT2B15 inhibitor amitriptyine (0–200 μM) was included as a positive control for UGT2B15. All incubations were performed in triplicate, and mean values were used for analysis.

### 4.4. K_i_ Determination of βC on the UGT1A1 Activity

Based on the lowest IC_50_ value, the inhibitor constant (K_i_) values of the βC for UGT1A1 were determined. Briefly, β-estradiol, a specific substrate for UGT1A1, was incubated with βC or nilotinib, a well-known UGT1A1 inhibitor. For determination of K_i_ values, β-estradiol concentrations used were 5, 10 and 20 μM. The concentrations of nilotinib and βC were as follows: 0–2 μM and 0–50 μM, respectively. All incubations were performed in triplicate, and the mean values were used for the analysis. Other procedures were similar to those of the reversible inhibition studies.

### 4.5. LC-MS/MS Analysis

The metabolites of the seven UGT-selective substrates were analyzed using an API 3200 triple quadrupole mass spectrometer (AB Sciex, Foster City, CA, USA) equipped with an Agilent 1260 HPLC system (Agilent Technologies, Wilmington, DE, USA) operating in electrospray ionization interface mode, as described previously [32,40]. Chromatographic separations were performed on an Agilent Poroshell 120 EC-C18 column (50 mm × 4.6-mm i.d.; 2.7-μm particle size; Agilent Technologies) to quantify β-estradiol-3-glucuronide (UGT1A1), chenodeoxycholic acid 24-acyl-β-d-glucuronide (UGT1A3), trifluoperazine-*N*-glucuronide (UGT1A4), propofol-*O*-glucuronide (UGT1A9), zidovudine-5′-glucuronide (UGT2B7), and 4-methylumbelliferyl glucuronide (UGT2B15); an Agilent Poroshell 120 EC-C18 column (100 × 4.6-mm i.d.; 2.7-mm particle size) was used for quantification of serotonin-*O*-glucuronide (UGT1A6). The isocratic mobile phase consisted of distilled water (A) and acetonitrile containing 0.1% formic acid (B) at a ratio (*v/v*) of 10:90 for chenodeoxycholic acid 24-acyl-β-d-glucuronide, 30:70 for trifluoperazine-*N*-glucuronide and zidovudine-5′-glucuronide, 50:50 for propofol-*O*-glucuronide and 4-methylumbelliferyl glucuronide, 65:35 for β-estradiol-3-glucuronide, and 90:10 for serotonin-*O*-glucuronide, at a flow rate of 0.5 mL/min. The total run time was 4.0 min per sample for each metabolite, with the exception of serotonin-*O*-glucuronide (6.0 min). Peak areas for all of the analytes were automatically integrated using Analyst software (version 1.5.2, Applied Biosystems, Foster City, CA, USA).

Three-day validations were performed to confirm the effectiveness of the LC-MS/MS system for determination of each of the six UGT-selective substrate metabolites in microsomal incubation mixtures, and 4-methylumbelliferyl glucuronide in recombinant UGT2B15 supersomes. The calibration standards were prepared at six concentrations (0.01–50 µM for each metabolite) in blank microsomal or recombinant UGT2B15 incubation mixtures. We found that the precision (≤13.7%) and accuracy (87.1%–111.7%) values were within acceptable ranges; our method was thus both reliable and reproducible.

### 4.6. Data Analysis

To determine IC_50_ values, the activities of each UGT isozyme in the presence of different concentrations of the five xanthophylls were compared with those of negative control incubations (containing no inhibitor; 0 μM). The IC_50_ values were calculated via nonlinear least-squares regression analysis from plots of the logarithms of inhibitor concentration vs. the residual percentages of activities after inhibition, using WinNonlin (ver. 4.0; Pharsight, Mountain View, CA, USA). The apparent inhibitory constants (the K_i_ values) were estimated from fitted curves using the WinNonlin software. Inhibition data were fitted to different models of enzyme inhibition (competitive, non-competitive, uncompetitive, or mixed) using nonlinear least-squares regression analysis (WinNonlin software). The most appropriate inhibition model was selected based on goodness-of-fit criteria determined via visual inspection, by calculating correlations of determination (R^2^ values), and using the corrected Akaike′s Information Criterion. For visual inspection, data were presented as Dixon plots.

## Figures and Tables

**Table 1:** IC_50_ values of known potent UGTs inhibitors and AS, βC, CA, LU and ZE towards the seven UGTs isoforms.

UGTs	IC_50_ Values (μM)						
	Known Potent Inhibitors		AS	βC	CA	LU	ZE
UGT1A1	Nilotinib	1.55 ± 0.118	>50 ^1^	18.8 ± 2.07	38.5 ± 4.65	45.5 ± 4.01	>50
UGT1A3	Deoxyschizandrin	5.17 ± 0.700	>50	28.3 ± 4.40	41.2 ± 3.14	>50	>50
UGT1A4	Hecogenin	8.07 ± 2.60	>50	34.9 ± 5.98	>50	28.7 ± 3.79	>50
UGT1A6	Diclofenac	106 ± 26.6	>50	>50	>50	>50	>50
UGT1A9	Niflumic acid	0.390 ± 0.0256	>50	>50	>50	>50	>50
UGT2B7	Efavirenz	37.8 ± 2.37	>50	>50	>50	>50	>50
UGT2B15 ^2^	Amitriptyline	69.4 ± 10.6	>50	>50	>50	>50	>50

IC_50_, 50% inhibitory concentration; AS, astaxanthin; βC, β-cryptoxanthin; CA, canthaxanthin; LU, lutein; ZE, zeaxanthin. ^1^ The residual activities at the highest tested xanthophylls concentration (50 μM) were greater than 50%. ^2^ Recombinant UGT2B15 supersomes was used. Data represent the mean ± standard deviation of triplicate determinations.

## Supplementary Materials

Supplementary materials can be accessed at: http://www.mdpi.com/1420-3049/21/8/1052/s1.

## Abbreviations

The following abbreviations are used in this manuscript:

UGT	UDP-glucuronosyltransferase
AS	astaxanthin
βC	β-cryptoxanthin
CA	canthaxanthin
LU	lutein
ZE	zeaxanthin
IC_50_	the 50% inhibitory concentration
K_i_	inhibitory constant
LC-MS/MS	liquid chromatography/tandem mass spectrometry
C_max_	the maximum plasma concentration
