# Supplementary material for: In Vitro Inhibition of Human UDP-Glucuronosyl-Transferase (UGT) Isoforms by Astaxanthin, β-Cryptoxanthin, Canthaxanthin, Lutein, and Zeaxanthin: Prediction of in Vivo Dietary Supplement-Drug Interactions
Source: Molecules. 2016 Aug 12;21(8):1052. doi: 10.3390/molecules21081052 (PMC6272861; doi:10.3390/molecules21081052)
Supplement: Supplementary file 1 [file molecules-21-01052-s001.pdf]

Supplementary Materials: In Vitro Inhibition of Human UDP-Glucuronosyltransferase (UGT) Isoforms by Astaxanthin, β-Cryptoxanthin, Canthaxanthin, Lutein, and Zeaxanthin: Prediction of in Vivo Dietary Supplement-Drug Interactions

Yu Fen Zheng, Jee Sun Min, Doyun Kim, Jung Bae Park, Sung-Wook Choi, Eun Seong Lee, Kun Na and Soo Kyung Bae

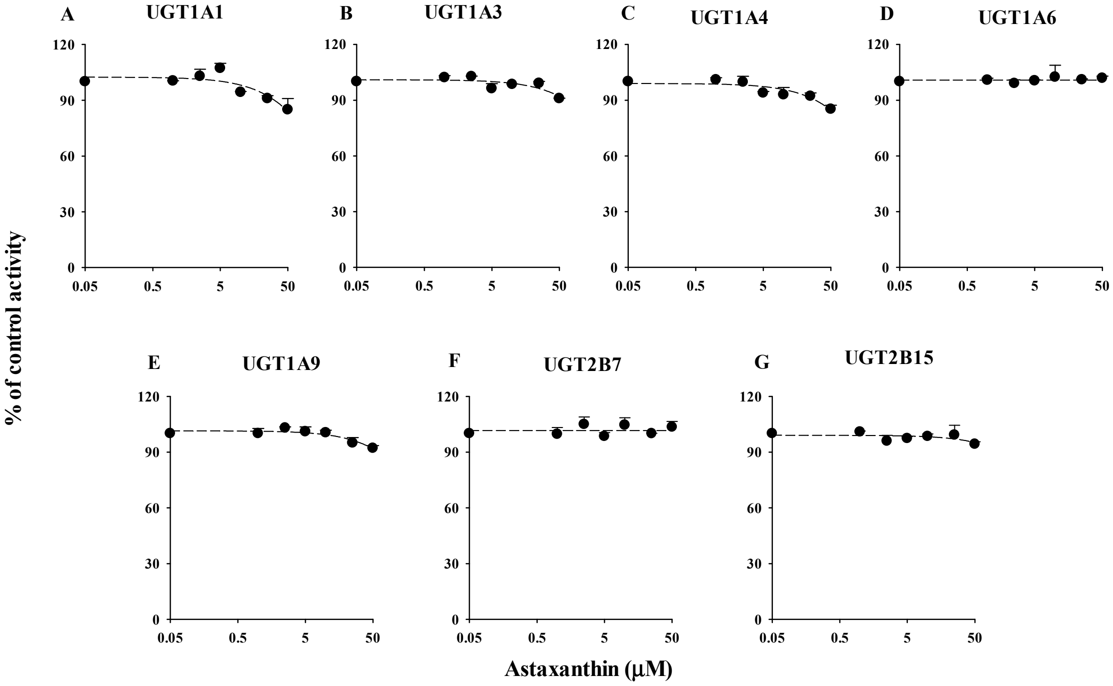

Figure S1. IC_{50} curves of astaxanthin for human UGTs activities including UGT1A1 for β-estradiol-3-glucuronidation (**A**); UGT1A3 for chenodeoxycholic acid 24-acyl-β-D-glucuronidation (**B**); UGT1A4 for trifluoperazine-*N*-glucuronidation (**C**), UGT1A6 for serotonin-*O*-glucuronidation (**D**); UGT1A9 for propofol-*O*-glucuronidation (**E**); and UGT2B7 for zidovudine-5′-glucuronidation (**F**) in human liver microsomes, and UGT2B15 for 4-methylumbelliferyl glucuronidation (**G**) in recombinant human UGT2B15 supersomes. Data are the mean ± standard deviation of triplicate determinations. The dashed lines represent the best fit to the data using non-linear regression.

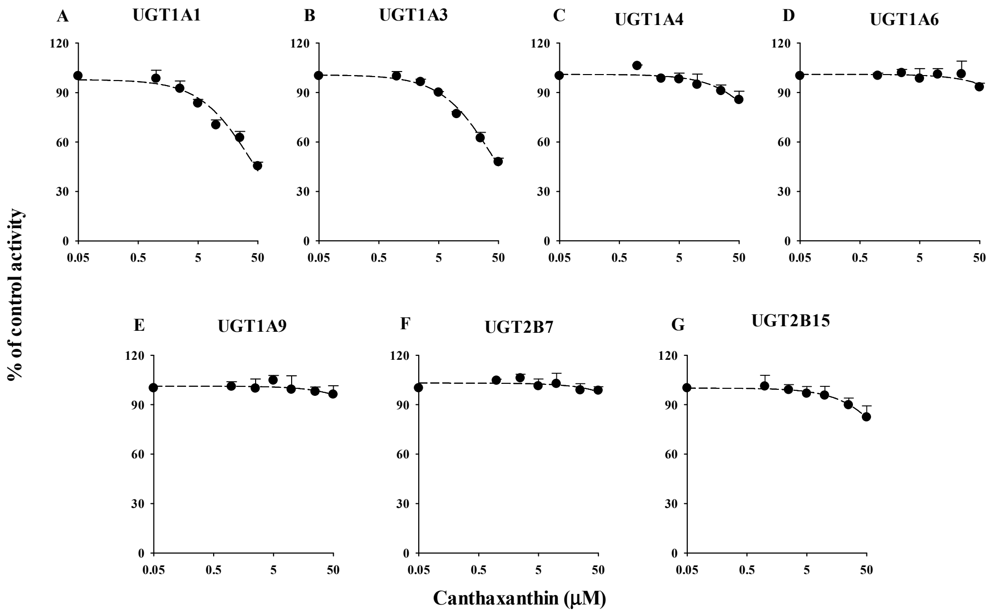

Figure S2. IC$_{50}$ curves of canthaxanthin for human UGTs activities including UGT1A1 for β-estradiol-3-glucuronidation (**A**); UGT1A3 for chenodeoxycholic acid 24-acyl-β-D-glucuronidation (**B**); UGT1A4 for trifluoperazine-*N*-glucuronidation (**C**); UGT1A6 for serotonin-*O*-glucuronidation (**D**); UGT1A9 for propofol-*O*-glucuronidation (**E**); and UGT2B7 for zidovudine-5′-glucuronidation (**F**) in human liver microsomes, and UGT2B15 for 4-methylumbelliferyl glucuronidation (**G**) in recombinant human UGT2B15 supersomes. Data are the mean ± standard deviation of triplicate determinations. The dashed lines represent the best fit to the data using non-linear regression.

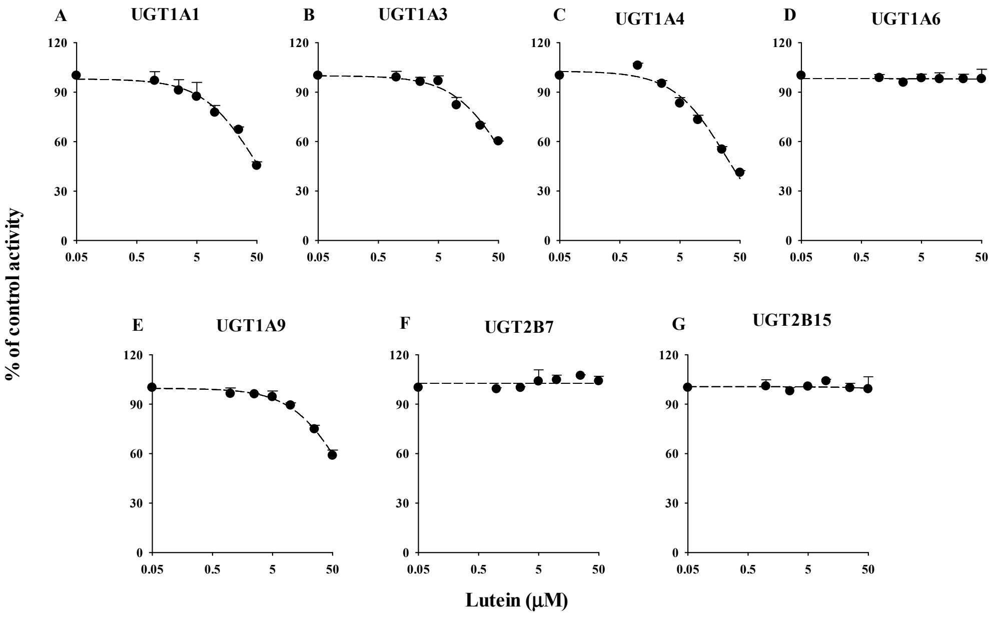

Figure S3. IC$_{50}$ curves of lutein for human UGTs activities including UGT1A1 for β-estradiol-3-glucuronidation (**A**); UGT1A3 for chenodeoxycholic acid 24-acyl-β-D-glucuronidation (**B**); UGT1A4 for trifluoperazine-*N*-glucuronidation (**C**); UGT1A6 for serotonin-*O*-glucuronidation (**D**); UGT1A9 for propofol-*O*-glucuronidation (**E**); and UGT2B7 for zidovudine-5′-glucuronidation (**F**) in human liver microsomes, and UGT2B15 for 4-methylumbelliferyl glucuronidation (**G**) in recombinant human UGT2B15 supersomes. Data are the mean ± standard deviation of triplicate determinations. The dashed lines represent the best fit to the data using non-linear regression.

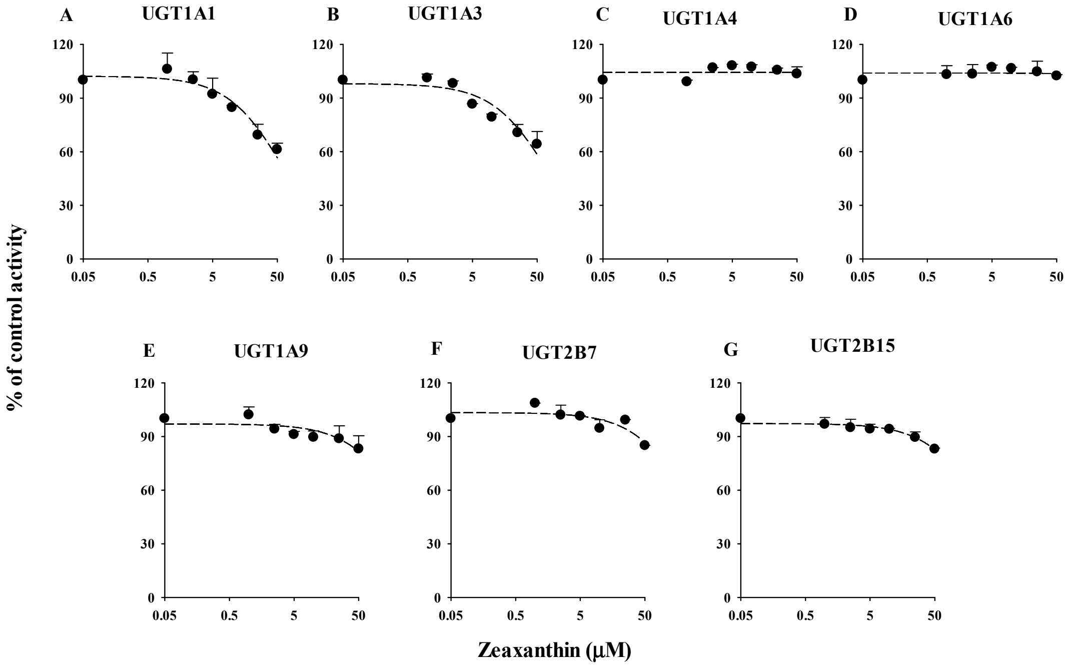

Figure S4. IC_{50} curves of zeaxanthin for human UGTs activities including UGT1A1 for β-estradiol-3-glucuronidation (**A**); UGT1A3 for chenodeoxycholic acid 24-acyl-β-D-glucuronidation (**B**); UGT1A4 for trifluoperazine-*N*-glucuronidation (**C**); UGT1A6 for serotonin-*O*-glucuronidation (**D**); UGT1A9 for propofol-*O*-glucuronidation (**E**); and UGT2B7 for zidovudine-5′-glucuronidation (**F**) in human liver microsomes, and UGT2B15 for 4-methylumbelliferyl glucuronidation (**G**) in recombinant human UGT2B15 supersomes. Data are the mean ± standard deviation of triplicate determinations. The dashed lines represent the best fit to the data using non-linear regression.
